# Microencapsulation of *Cyclocarya paliurus* (Batal.) Iljinskaja Extracts: A Promising Technique to Protect Phenolic Compounds and Antioxidant Capacities

**DOI:** 10.3390/foods10122910

**Published:** 2021-11-24

**Authors:** Xiao Chen, Senghak Chhun, Jiqian Xiang, Pipat Tangjaidee, Yaoyao Peng, Siew Young Quek

**Affiliations:** 1 Food Science, School of Chemical Sciences, The University of Auckland, Auckland 1010, New Zealand; xche622@aucklanduni.ac.nz (X.C.); schh883@aucklanduni.ac.nz (S.C.); ptan226@aucklanduni.ac.nz (P.T.); yaoyao.peng@auckland.ac.nz (Y.P.); 2 Enshi Tujia & Miao Autonomous Prefecture Academy of Agricultural Sciences, Enshi 445002, China; hmxjq@163.com; 3 Riddet Institute, Centre of Research Excellence in Food Research, Palmerston North 4474, New Zealand

**Keywords:** spray drying, gum acacia, maltodextrin, plant extract, functional ingredients, powder

## Abstract

This study aimed to protect phenolic compounds of *Cyclocarya paliurus* (Batal.) Iljinskaja (*C. paliurus*) using a microencapsulation technique. Ethanol and aqueous extracts were prepared from *C. paliurus* leaves and microencapsulated via microfluidic-jet spray drying using three types of wall material: (1) maltodextrin (MD; 10–13, DE) alone; (2) MD:gum acacia (GA) of 1:1 ratio; (3) MD:GA of 1:3 ratio. The powders’ physicochemical properties, microstructure, and phenolic profiles were investigated, emphasizing the retentions of the total and individual phenolic compounds and their antioxidant capacities (*AOC*) after spray drying. Results showed that all powders had good physical properties, including high solubilities (88.81 to 99.12%), low moisture contents (4.09 to 6.64%) and low water activities (0.11 to 0.19). The extract type used for encapsulation was significantly (*p* < 0.05) influenced the powder color, and more importantly the retention of total phenolic compounds (TPC) and *AOC*. Overall, the ethanol extract powders showed higher TPC and *AOC* values (50.93–63.94 mg gallic acid equivalents/g and 444.63–513.49 µM TE/g, respectively), while powders derived from the aqueous extract exhibited superior solubility, attractive color, and good retention of individual phenolic compounds after spray drying. The high-quality powders obtained in the current study will bring opportunities for use in functional food products with potential health benefits.

## 1. Introduction

*Cyclocarya paliurus* (Batal.) Iljinskaja (*C. paliurus*), also named “sweet tea tree”, is an edible and medicinal plant native to southern China, predominantly Jiangsu, Hunan, Hubei, Henan, Guangxi, Fujian, and Anhui provinces [1]. *C. paliurus* leaves were approved by the Food and Drug Administration as the first Chinese health tea in 1999 [2]. This is because the extract of *C. paliurus* exhibits numerous health benefits, ranging from antihyperlipidemic and antihyperglycemic [3] to antioxidant [3,4] and antimicrobial activities [5,6]. Many other therapeutic effects were also observed for *C. paliurus*, for example, immunomodulation, antihypertensive activity, and improvement of mental efficiency [1,7,8]. These bioactivities are associated with the presence of abundant phenolic compounds in leaves, such as myricetin-3-*O*-*β*-d-glucuronide, kaempferol, quercetin, caffeic acid, quercetin-3-*O*-*β*-d-glucuronide, and quercetin-3-*O*-*α*-d-glucuronide [5,9]. Other phytochemical compounds, for example polysaccharides and triterpenoids, also show antidiabetic, anticancer, anti-inflammatory, and hepatoprotective properties [10,11,12,13]. 

Bioactive ingredients can be extracted from plants and further incorporated into different foods for developing functional products. However, the application of plant-derived phenolic compounds, especially polyphenols, is limited by their low stability as induced by pH, temperature, oxygen, light and enzymes [14]. Microencapsulation of plant extracts by spray-drying is among the viable solutions, in which bioactive components can be entrapped by wall materials and thereby well protected from any harsh environmental conditions. Moreover, microencapsulation of *C. paliurus* extracts into powders brings numerous other advantages such as ease of storage, transportation, and application in foods. Regarding the drying technique, conventional spray-drying is inferior to the monodisperse droplet technique in terms of controlling the physicochemical and functional properties of the resulting powders [15]. This is because traditional spray-drying exhibits the drawback of producing atomized droplets of diverse sizes, which means the droplets will experience different drying histories even under the same drying conditions [15]. By contrast, the micro-fluidic-jet spray dryer resembles a special nozzle that aids in the production of microcapsules with uniform morphology, shape and particle size [15,16]. Up to now, this novel technique has been successfully used in the production of spray-dried powders including fish oil emulsion [17], vitamin E and CoQ_10_ [18,19], cranberry juice [20], noni juice [16], and mandarin juices [21], but has yet been explored for plant extracts.

Maltodextrin (MD) and gum acacia (GA) are popular hydrocolloid-based materials for encapsulating sensitive components or fruit/vegetable juices, such as green tea polyphenols [22], spent coffee’s phenolic extract [23], *Phaseolus lunatus* bioactive peptides [24], fermented noni juice [25], extracted soy isoflavones [26], and mandarin juice [21]. The wide use of GA is due to its high solubility, ideal emulsifying properties and good viscosity [27]. On the other hand, MD has fast reconstitution ability, which creates colorless and low viscous solutions without altering the original flavor properties of the core material [28]. Some studies have shown that blends of GA and MD are more efficient than a single carrier during microencapsulation, which significantly increases the stability of bioactive compounds in food systems [16,20]. 

Nowadays, tea infusion is one of the main food-level usages of *C. paliurus*. To diversify the application of *C. paliurus* in food products and ensure the stability of its bioactive extracts, microencapsulation of *C. paliurus* into a solid form is favorable, but no study has been conducted so far, to the best of the authors’ knowledge. This study aimed to encapsulate the ethanol and aqueous extracts of *C. paliurus* via a monodisperse spray-dryer using MD and its combination with GA as wall materials. Physicochemical properties of spray-dried powders were studied, emphasizing on the retentions of total and individual phenolic compounds and their *AOC* after spray drying. This study filled the knowledge gap in the spray-drying of *C. paliurus* extracts while offering an alternative way to deliver bioactive components of *C. paliurus* to consumers.

## 2. Materials and Methods

### 2.1. Materials and Chemicals

Commercially dried *C. paliurus* leaves were supplied by the Enshi Tujia & Miao Autonomous Prefecture Academy of Agricultural Sciences, Hubei province, China. The leaves were ground into a powder using a coffee blender (Breville, Australia) for 3 min, and subjected to a 200 mesh US standard sieve to achieve a small and uniform particle size that facilitates the extraction. The fine powder samples were kept at −20 °C until further requirement. MD [10,11,12,13], dextrose equivalent (DE) was purchased from Ingredion (Singapore) and GA was gifted by Hawkins Watts Limited (Auckland, New Zealand).

Folin-Ciocalteu’s phenol reagent, gallic acid, 2,4,6-tri(2-pyridyl)-s-triazine (TPTZ), 2,2-diphenyl-1-picrylhydrazyl (DPPH), and 6-hydroxy-2,5,7,8-tetramethyl-chroman-2-carboxylic acid (Trolox) were acquired from Sigma-Aldrich (St. Louis, MO, USA). Authentic standards including kaempferol-3-*O*-glucoside, kaempferol-3-*O*-rhamnoside, myricetin-3-*O*-galactoside, and quercetin-3-*O*-rhamnoside were purchased from Extrasynthese (Genay, France). Quercetin-3-*O*-galactoside and quercetin-3-*O*-glucuronide were sourced from Cayman Chemical (Ann Arbor, MI, USA). Phenolic acid standards including 1,3-dicaffeoylquinic acid, 5-*O*-caffeoylquinic acid, 4,5-dicaffeoylquinic acid, 3,4-dicaffeoylquinic acid, and 1,5-dicaffeoylquinic acid were obtained from Biosynth Carbosynth® (Carbosynth Ltd., Berkshire, UK). Other phenolic compounds including quercetin-3-*O*-glucoside, caffeic acid, chlorogenic acid, and phloretin were acquired from Sigma-Aldrich (St. Louis, MO, USA).

### 2.2. Preparation of C. paliurus Extracts

Experiments were conducted to optimize the extraction parameters, including time, temperature, material to solvent ratio (*w*/*v*), and ethanol concentration (%), using orthogonal design (data not shown). The optimum conditions were then applied to prepare the ethanol and aqueous extracts as below. For the preparation of ethanol extract, 1 g of ground sample was placed into an Eppendorf tube, followed by a 30% ethanol solution supplement to achieve a material to solvent ratio of 1:30 (*w*/*w*). The extraction was carried out at 60 °C for 60 min with constant stirring at 550 rpm. Afterwards, the solution was vacuum filtered with a MS2 filter paper (MicroScience, New South Wales, Australia) and stored at −20 °C until further analysis. For the preparation of the aqueous extract, the procedure used was similar to that described above. The optimum conditions applied were material to solvent ratio of 1:30 (*w*/*w*) (1 g of ground sample, 30 mL of Mill-Q water), extraction at 90 °C, and extraction time of 60 min under constant stirring of 550 rpm.

### 2.3. Feed Solution and Spray-Drying Process

Preliminary experiments were conducted to find the suitable spray drying conditions and feed solution composition. According to the results, three formulations (Table 1) were chosen from each extract for preparing the feed solutions. The wall materials or carrier matrices were blended with either the ethanol or aqueous extracts of *C. paliurus* to achieve a core to wall ratio of 1:3 (*w*/*w*). The sample was thoroughly mixed at room temperature by stirring at 700 rpm using a magnetic hot plate until all solids were fully dissolved.

A micro-fluidic-jet spray-dryer supplied by Dong Concept Pty Ltd. (Nantong, China) was used to spray dry feed solutions [21]. The drying parameters were set as follows based on results from the preliminary experiments: inlet temperature of 180 °C, pressure at 0.5 ± 0.02 kg/cm^2^, cooling airflow rate at 250 ± 2 L/min, nozzle driver frequency of 10kHz, dispersed air flow rate at 10 L/min, reservoir temperature of 20 °C, and outlet temperature of 81 °C. A polytetrafluoroethylene nozzle with a diameter of 75µm was utilized to obtain monodisperse droplets during the drying process. Powders collected from the dryer were immediately transferred into sample tubes which were then flushed with nitrogen, sealed by parafilm and stored in a desiccator kept at 4 °C before further analysis. 

### 2.4. Characterization of Spray-Dried Powders

#### 2.4.1. Moisture Content and Water Activity

The water activity (a_w_) was measured using a water activity analyzer (Novasina, AW SPRINT, Switzerland) at 25 °C. The moisture content of powder samples was determined by the gravimetry method, by drying the samples in an oven (Heraeus D-63450, Germany) at 105 °C until a consistent weight was maintained.

#### 2.4.2. Color Analysis

The color of powder samples was evaluated using three indices (a*, b*, and L*) obtained from a colorimeter (Minolta CR-300, Tokyo, Japan). The a* value denotes the variation from red (60) to green (−60), the L* value indicates lightness from blank (0) to white (100), and the b* value represents the variation from yellow (60) to blue (−60).

#### 2.4.3. Bulk Density

Bulk (ρbulk) density was obtained as described in our previous study [21]. Briefly, spray-dried *C. paliurus* powders were accurately weighted and gently put into a 10 mL cylinder. The powder volume (*V*_0_) was recorded and the ρbulk was calculated using Equation (1).

(1) $$\rho_{bulk}\;\left(g/\mathrm{mL}\right)=\frac{powder\;mass}{V_{0}}$$

#### 2.4.4. Hygroscopicity 

Powder hygroscopicity was measured according to Rigon & Noreña [29] with minor modifications. Briefly, 300 mg powder was put into an aluminium cap and then transferred into a chamber containing saturated NaCl with roughly 75% relative humidity at room temperature. The sample mass was accurately taken after 48 h of storage. The hygroscopicity was calculated as the mass of moisture (g) absorbed by 100 g powder (g/100 g). 

#### 2.4.5. Solubility

The solubility of powders was measured as described in our previous study [21]. In brief, 0.75 g sample was resuspended in 75 mL of Milli-Q water under vortex for 5 min. The suspension was centrifuged at 7000× *g* for 10 min. An aliquot of 20 mL supernatant was transferred into a pre-weighed beaker and oven-dried (Heraeus D-63450, Hanau, Germany) at 105 °C until a consistent weight was achieved. Solubility (%) was determined by the mass difference following Equation (2).

(2) $$\mathrm{Solubility}\;\left(\%\right)=\frac{\left(M_{1}-M_{0}\right)}{0.2}\times 100$$

where $M_{0}$ is the baker mass, and $M_{1}$ is the total mass of the sample and beaker after drying.

### 2.5. Particle Morphology

The powder morphology was visualized using a Hitachi Scanning Electron Microscope (Mode TM3030Plus, Tokyo, Japan) as described [16,25]. Briefly, the powder sample was placed on a carbon adhesive tape stuck on an aluminium stub. The sample was sputter-coated with a golden layer and then transferred into the specimen chamber for observation. The surface, overall appearance, and cross-section of the powder were observed under 500–800× magnification.

### 2.6. Total Phenolic Content (TPC) Analysis

TPC of the powder samples was obtained using the Folin-Ciocalteu assay [16,20]. Gallic acid (GA) solutions were prepared at a series of concentrations to construct a standard curve (R^2^ > 0.99). Results were expressed as mg GA equivalents/g dried powders [20]. 

The retention of TPC after spray drying was calculated using Equation (3).

(3) $$\mathrm{TPC}\;\mathrm{retention}\;\left(\%\right)=\frac{\mathrm{TPC}\;\mathrm{in}\;\mathrm{powder}}{\mathrm{Total}\;\mathrm{solids}\;\mathrm{content}\;\mathrm{in}\;\mathrm{powder}}÷\frac{\mathrm{TPC}\;\mathrm{in}\;\mathrm{feed}\;\mathrm{solution}}{\mathrm{Total}\;\mathrm{solids}\;\mathrm{content}\;\mathrm{in}\;\mathrm{feed}\;\mathrm{solution}}\times 100\;$$

### 2.7. Antioxidant Capacity (AOC) Analysis

#### 2.7.1. DPPH Assay

The DPPH assay was performed according to the procedure described by Lei et al. with minor modifications [5]. Briefly, the sample (10 μL) was transferred into a 96-well plate and 200 μL of a methanolic DPPH solution (40 mg/mL) were added. The reaction took place in darkness for 60 min. Afterwards, the sample absorbance was measured at 517 nm using a plate reader (EnSpire Multimode, Perkin Elmer, Waltham, MA, USA). The standards and the reagent blank were ethanolic Trolox (at 10, 25, 50, 100, 200, 300, and 400 µM) and absolute ethanol, respectively. Results were expressed as Trolox equivalents per g dried powders (µM TE/g dw). 

The retention of *AOC* as evaluated by the *DPPH* assay was calculated using Equation (4).

(4) $$AOC\;\mathrm{retention}\;\left(\%\right)=\frac{{AOC}_{DPPH}\;\mathrm{in}\;\mathrm{powder}}{\mathrm{Total}\;\mathrm{solids}\;\mathrm{content}\;\mathrm{in}\;\mathrm{powder}}÷\frac{{AOC}_{DPPH}\;\mathrm{in}\;\mathrm{feed}\;\mathrm{solution}}{\mathrm{Total}\;\mathrm{solids}\;\mathrm{content}\;\mathrm{in}\;\mathrm{feed}\;\mathrm{solution}}\times 100\;$$

#### 2.7.2. Ferric Reducing Antioxidant Powder (FRAP) Assay

An aliquot of 10μL sample was mixed with 200 μL of FRAP working solution [23]. The working solution was made up of the following constituents in a proportion of 10:1:1 (*v/v*), i.e., sodium acetate buffer (0.3 M, pH 3.6), 20mM FeCl_3_ solution, and 10 mM TPTZ diluted in 40 mM HCl. After incubation in the dark for 60 min, the sample was submitted to the EnSpire Multimode plate reader (Perkin Elmer, USA) for absorbance analysis at 593nm. A calibration curve (R^2^ > 0.99) was constructed using ethanolic Trolox solutions of 10, 25, 50, 100, 200, 300, and 400 µM. The *AOC* were expressed as Trolox equivalents (TE) per g dried powders (µM TE/g dw).

The retention of *AOC* as evaluated by the FRAP assay was calculated using Equation (5).

(5) $$AOC\;\mathrm{retention}\;\left(\%\right)=\frac{{AOC}_{FRAP}\;\mathrm{in}\;\mathrm{powder}}{\mathrm{Total}\;\mathrm{solids}\;\mathrm{content}\;\mathrm{in}\;\mathrm{powder}}÷\frac{{AOC}_{FRAP}\;\mathrm{in}\;\mathrm{feed}\;\mathrm{solution}}{\mathrm{Total}\;\mathrm{solids}\;\mathrm{content}\;\mathrm{in}\;\mathrm{feed}\;\mathrm{solution}}\times 100\;$$

### 2.8. Individual Phenolic Compound Analysis

Phenolic compounds in the feed solutions and spray-dried powders were identified and quantitated by an Agilent high-performance liquid chromatography (HPLC) system equipped with a Phenomenex C_18_ column (5 μm, 4.6 × 250 mm, Torrance, CA, USA) and a diode array detector. Mobile phases of (A) acetonitrile with 0.1% (*v/v*) formic acid and (B) water with 0.1% (*v/v*) formic acid were used at the flow rate of 1mL/min under 25 °C for compound separation. The gradient elution was conditioned as follows: 0min, 5% B; 10 min, 15% B; 20 min, 25% B; 30 min, 35% B; 35 min, 45% B; 40 min, 35% B; 45 min, 15% B; and 50 min, 5% B. The detector wavelength was set as 280 and 320 nm. 

The stock solution containing 14 phenolic standards was used to identify and quantitate analytes in the present study, including quercetin-3-*O*-rhamnoside, quercetin-3-*O*-glucuronide, quercetin-3-*O*-glucoside, 4,5-dicaffeoylquinic acid, quercetin-3-*O*-galactoside, kaempferol-3-*O*-glucoside, kaempferol-3-*O*-rhamnoside, myricetin-3-*O*-galactoside, 1,5-dicaffeoylquinic acid, caffeic acid, 5-*O*-caffeoylquinic acid, 3,4-dicaffeoylquinic acid, chlorogenic acid, and 1,3-dicaffeoylquinic acid. The stock solation was diluted into a series of concentrations to construct standard curves (see Supplementary Table S1). The retention of individual phenolic compounds after spray drying was calculated using Equation (6).

(6) $$\mathrm{Phenolic}\;\mathrm{retention}\;\left(\%\right)=\frac{\mathrm{Content}\;\mathrm{in}\;\mathrm{powder}}{\mathrm{Total}\;\mathrm{solids}\;\mathrm{content}\;\mathrm{in}\;\mathrm{powder}}÷\frac{\mathrm{Content}\;\mathrm{in}\;\mathrm{feed}\;\mathrm{solution}}{\mathrm{Total}\;\mathrm{solids}\;\mathrm{content}\;\mathrm{in}\;\mathrm{feed}\;\mathrm{solution}}\times 100\;$$

### 2.9. Statistical Analysis

All experiments were conducted in triplicate, and data were presented as mean ± standard deviation. One-way ANOVA and Duncan’s multiple range test was carried out to evaluate statistical significance using SPSS Statistics 25 from IBM (Armonk, NY, USA). The principal component analysis (PCA) was conducted using SIMCA 16.0 software from Sartorius (Umeå, Sweden).

## 3. Results and Discussion

### 3.1. Physical Properties of Spray-Dried C. paliurus Extracts

#### 3.1.1. Water Activity and Moisture Content

Moisture content indicates the water available in a food system and is an important factor determining the powder products’ stability and shelf life. The food industry prefers a value ranging from 1–6% for a stable storage purpose [20]. From Table 2, except for the EE-MD/GA-3 sample (6.64%), all powders fell in this range, i.e., from 4.09–5.95%, suggesting good stability of spray-dried *C. paliurus* extracts during storage. The use of MD as wall material without GA decreased moisture residue in the final product, which was evident for the *C. paliurus* powders derived from the ethanol extract (*p <* 0.05). A similar influence was found during the microencapsulation of *Sideritis stricta* tea [30], aҫai berry [31], and cranberry juice [20]. Since GA has high number of hydrophilic pockets associated with water-binding effect, more considerable water residue could be retained in the GA-encapsulated powders [20]. In addition, residue protein (2%) in GA could also increase the percentage of bound water inside the molecules, leading to a lower water diffusion rate during processing [32].

Water activity (a_w_) represents the free water available for biochemical and microbial reactions. As an essential index for spray-dried powders, water activity largely influences product safety and shelf life. Powders with an a_w_ below 0.6 could be microbiologically stable due to the limited availability of free water molecules for microbial proliferation [33]. The current samples exhibited the a_w_ values ranging from 0.11 to 0.19, regardless of wall materials and extract types. These a_w_ values were much lower than the powders produced from other plant-originated materials, e.g., watermelon [33], cranberry juice [20], and aҫai berry [31]. Our results indicated that the spray-dried *C. paliurus* extracts were microbiologically stable. Furthermore, the use of MD as wall material alone significantly lowered the water activity of the final products (*p <* 0.05), in line with the phenomenon of moisture-content reduction as discussed above.

#### 3.1.2. Hygroscopicity

Hygroscopicity indicates the vulnerability of a powder product to absorb moisture when exposed to high relative humidity conditions. In the present study, all samples showed low hygroscopicity ranging from 2.07–3.70% in an environment having 75% relative humidity (Table 2). The values were much lower than those reported for fruit juice powders produced from noni (19.4–22.7%) [16], aҫai berry (11.8–19.7%) [31], and mandarin (17.5–18.53%) [21]. For food powders, higher hygroscopicity is frequently associated with core material rich in sugars and organic acids with low glass transition temperatures, which increase the powder’s tendency to water adsorption [34]. The hygroscopicity results obtained from this research generally agreed with that of the spray-dried grape polyphenols extract encapsulated by MD:GA of 6:4 under the core to coating ratio of 1:2 (hygroscopicity, 2.5%), as reported by Tolun et al. [35]. They also found that the type of wall materials applied has a profound effect on the powder hygroscopicity. For example, MD with a relatively higher DE (17–20) could raise the hygroscopicity value of the resulting powder, probably due to the presence of more hydroxyl moieties.

In the current study, the powders produced from both the ethanol and aqueous extracts of *C. paliurus* showed no significant variations in hygroscopicity regardless of the wall material used. However, the hygroscopicity (3.00–3.70%) of the aqueous extract powders was significantly (*p <* 0.05) higher than all the ethanol extract samples (2.07–2.16%). These results indicate that the nature of the extract used for feed solution preparation could have a more significant effect on the powders’ hygroscopicity than the wall materials applied. Nevertheless, the effect of wall material on powder hygroscopicity cannot be dismissed. In a previous study, we applied similar wall materials, i.e., GA and MD (two types, 17–20 DE and 10–13 DE), for encapsulation of noni juice, and found the wall material type significantly affected the hygroscopicity of the powders produced [16]. The trend of powder hygroscopicity was observed as MD (17–20 DE) > GA > MD (10–13 DE). In the current study, we have applied a combination of MD and GA in different ratios instead of using a single wall material. The interaction of MD and GA would modify the hydrogen bonds of the hydroxyl groups in the crystalline and amorphous regions of macromolecules, affecting the moisture absorption ability of the wall materials [16]. This may be the reason for the different results obtained using single wall material compared to when used in combination.

#### 3.1.3. Solubility

Solubility, reflecting the reconstitution capacity of powders, is an essential indicator for assessing the quality of spray-dried powders. High solubility is preferred by the food industry for the manufacturing of different food products. Overall, the solubility of the powder samples is high within the range of 88.8% to 99.12% (Table 2). The aqueous extract microencapsulated using MD (WE-MD-1) gave the maximum solubility (99.12%) among the powders. In comparison with previous studies, the solubility of spray-dried powders derived from *C. paliurus* extracts was significantly superior to that of the mandarin juice (73.82–75.30%) [21], aҫai berry incorporated in Tapioca starch (32.08%) [31], and green tea leaf extracts (63–72.66%) [36]. The obtained solubility values were also comparable to that of the spray-dried powders containing mao fruit juice (89.29–96.87%) [37], and the aqueous extracts of mountain tea (97.3–98.6%) [30] and blackberry (88.2–97.4%) [29]. Comparing the two different types of extracts, the solubility of powders produced from the aqueous extract were significantly (*p* < 0.05) higher than those of the ethanol extracts (i.e., 92.04 to 99.12% vs. 88.81 to 89.85%). Silva et al. reported a similar finding for green tea microcapsules. They explained that chemicals with hydrophobic groups might present in the original ethanol extract, leading to lower solubility [36]. Their argument was supported by a study on a medicinal plant called Devil’s Club (*Oplopanax horridus*) where several hydrophobic constituents have been identified in the ethanol extract including *trans*-nerolidol, falcarindiol, oplopandiol acetate, and oplopandiol [38].

#### 3.1.4. Bulk Density

Bulk density is a crucial parameter for powder products as it is associated with mixing, packaging, transportation, and storage [30]. In the present study, the bulk densities ranged from 0.30 to 0.38 g/mL for all samples, comparable to that of the particles derived from the mountain tea water extract (0.38 g/mL) [30]. In contrast, the bulk density of mandarin juice powders (0.56–0.61 g/mL) [21] and avocado powders (0.41–0.51 g/mL) [32] were much higher. The differences observed may be ascribed to the particle size and surface morphology of the powders. For example, a large particle size with a wrinkled surface would result in a greater interspace between particles, therefore occupying a high volume, giving lower bulk density [16]. The particle size distribution of spray-dried powders could also be responsible for this phenomenon. A homogenous spray-dried powder with uniform particle size decreases bulk density due to larger interparticle voids. In contrast, in a system of non-uniform particle size, bulk density will increase because the spaces between large interparticle voids are loaded with smaller microcapsules [39]. Furthermore, the type of wall material used for microencapsulation could influence the surface structure of particles and consequently affect the bulk density of powders produced. For example, Zhang et al. found that the noni juice powders encapsulated by GA-alone had a shriveled and rough surface with thicker indentations [16]. This surface morphology causes a large interspace between particles, giving a lower bulk density. However, the effect of wall materials was not apparent in the current study, probably due to the use of MD and GA in combination instead of using each individually.

#### 3.1.5. Color Evaluation

Color gives the first sensory impression and quality about a food product. Table 3 shows the color attributes and appearance of different spray-dried microcapsules of *C. paliurus* ethanol and aqueous extracts. Generally, the L* value varied from 59.23 to 60.52. The b* and a* values showed a relatively wider range, from 14.82 to 19.07 and from11.49 to 13.68, respectively. Both wall materials and extracts used for encapsulation showed no significant effect on the brightness (L*). In contrast, the a* and b* values showed significant statistical differences; the *C. paliurus* aqueous extract powders were less reddish and more yellow than those obtained from the ethanol extract. Moreover, the wall material type significantly affected the redness (a*) and the yellowness (b*) of the powders (*p* < 0.05), with partial substitution of MD with GA in the feed solution producing more reddish and browner powders. Şahin Nadeem et al. [30] reported similar observations for spray-dried mountain tea extract. They ascribed this to the natural color of the core material and the minor protein content (2%) and reducing sugars in GA, which were susceptible to the Maillard browning reactions during spray-drying.

### 3.2. Morphology Observation

**Figure 1:**
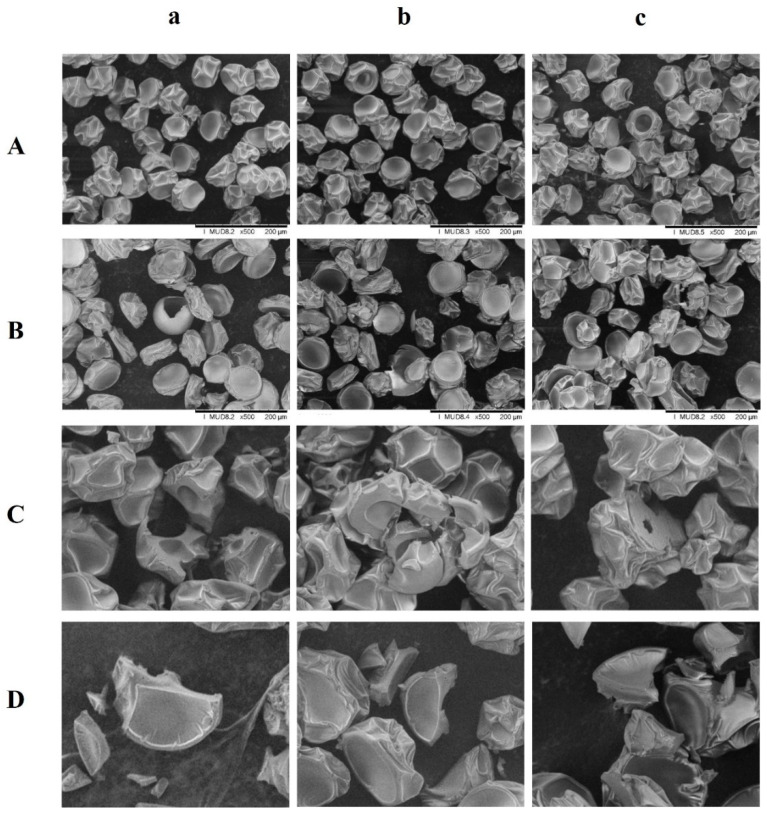
SEM micrograph of spray-dried *C. paliurus* extracts microencapsulated with (a) MD, (b) MD and GA at the ratio of 1:1, and (c) MD and GA at the ratio of 3:1. Rows (**A**,**B**) refers to the surface structure of powders containing aqueous and ethanol extract, respectively (500× magnification); rows (**C**,**D**) are the cross-section structure of the aqueous-extract and ethanol extract powders, respectively (800× magnification).

Figure 1 reveals the external and cross-section morphology of the spray-dried *C. paliurus* powders. Generally, all samples have a marginally spherical shape with size uniformity, surface wrinkles and dents, and no apparent cracks (rows A and B; columns a, b, and c). The wrinkle and dent formations on the particle surface may be caused by rapid water removal and cooling of the surface crust [35]. The intact particle surface is desirable as it minimizes gas permeability and reduces the surface area for oxidation, thereby enhancing the preservation and retention of the core material [16,35,36]. Moreover, the uniformity of particle shape and size observed for all samples was significantly different from the agglomerated and uneven morphology of powders prepared using the conventional spray-drying technique [30,31,32]. The consistent size and surface morphology was attributed to using a micro-fluidic-jet spray-dryer equipped with a special nozzle [15].

There were no clear correlations between surface morphology and wall materials used for the spray-dried powders from the aqueous extracts (rows A; columns a, b, and c). For the powders containing the ethanol extract (row B), the MD-GA particles (row B; columns b and c) displayed relatively more roughness and shaper indentations than the MD-alone powder (row B; columns a). Zhang et al. reported a similar phenomenon for the cranberry juice powders produced via a conventional mini spray dryer [20]. The rate of dry-shell formation determines the surface morphology due to the inflation and rupture of voids during the spray-drying process [40]. An early dry shell formation resulted in uneven moisture distribution within the particle, and consequently, a shriveled and rough surface [41]. Furthermore, the cross-section images (rows C and D; columns a, b, and c) also did not illustrate any obvious differences among the three samples with different wall materials compositions. However, the type of extract used for spray drying indicates an influence on the cross-section morphology of the final microcapsules. The surfaces of the ethanol extract particles (row D) were generally more homogenous with few smaller pores than the aqueous extract samples (row C). Whether this structure would be conducive to the retention of total phenolic compounds in the spray-dried powders will be further investigated in the following section.

### 3.3. Retentions of TPC and AOC after Spray Drying

**Figure 2:**
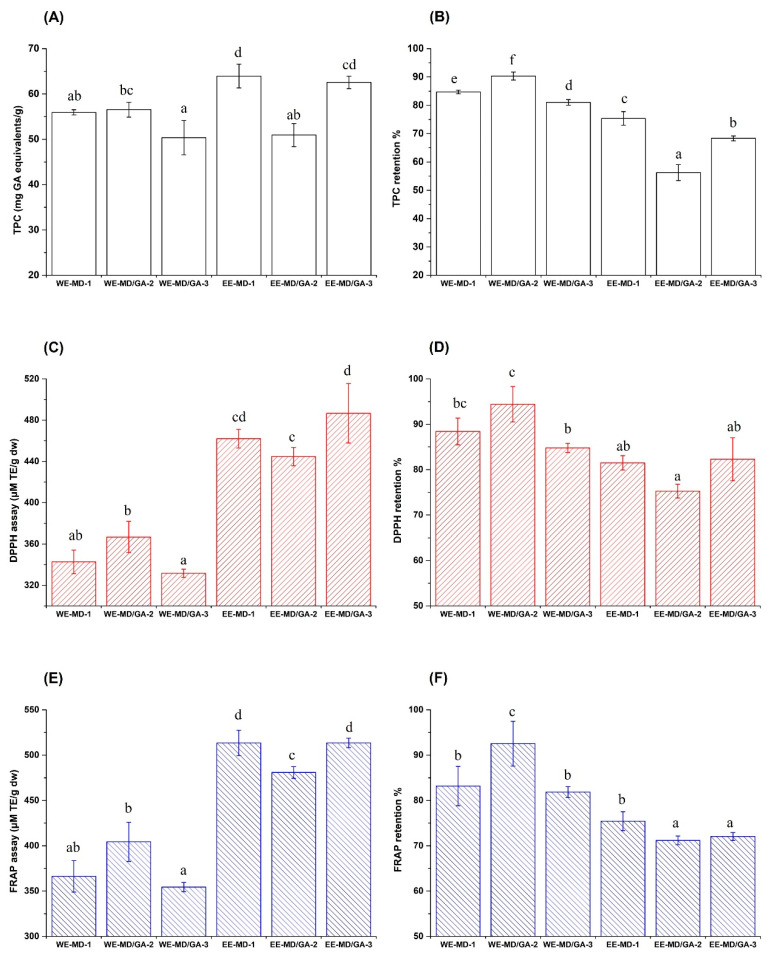
TPC and *AOC* of spray-dried *C. paliurus* extracts and their retentions after spray drying. (**A**) TPC of spray-dried powders; (**B**) The retention (%) of TPC after spray drying; (**C**) *AOC* of spray-dried powders evaluated by DPPH assay; (**D**) retention (%) of *AOC* after spray drying obtained by DPPH assay; (**E**) *AOC* of spray-dried powders evaluated by FRAP assay; (**F**) retention (%) of *AOC* after spray drying obtained by FRAP assay. (WE, aqueous extract; EE, ethanol extract; MD, malto-dextrin (10–13 DE); GA, gum acacia. The numbers indicate the wall materials ratio; 1 = only MD, 2 = MD:GA of 1:1 ratio, 3 = MD:GA of 1:3 ratio). Different letters (a, b, c, d, e, f) in the figure indicate significant differences at the level of *p* < 0.05.

TPC and *AOC* retentions were examined to evaluate the effect of spray-drying on the bioactive compounds in the *C. paliurus* extracts. The results are shown in Figure 2. Furthermore, the TPC and *AOC* values of the original aqueous and ethanol extracts are listed in Supplementary Table S2 for comparison.

The TPC of powder varied from 50.35 to 63.94mg GA equivalents/g, significantly lower than the value of the original aqueous and ethanol extracts (65.18 and 94.76 mg GA equivalents/dw). This indicated the degradation of some phenolic compounds during thermal treatment as induced by the spray drying process. Both wall material and extract types significantly (*p* < 0.05) influenced the TPC retention in the final products (Figure 2B). The highest TPC retention was in the WE-MD/GA-2 sample (90.31%), followed by the WE-MD-1 and WE-MD/GA-3 samples (84.69% and 81.02%, respectively). These results indicate that the MD-GA mixture in a 1:1 ratio worked well to protect the phenolic compounds of *C. paliurus* aqueous extract. The superiority of MD-GA blends was also observed for the microencapsulation of cranberry juice (TPC retention, 138–216%) [20] and mandarin juice (chlorogenic acid retention, 92.7–225%) [21], and was further confirmed by Idham et al. [42], who studied the microencapsulation efficiency of anthocyanins from *Hibiscus Sabdariffa* L. The chemical composition of MD and GA could have contributed to their superiority in preserving the TPC of the aqueous extract-derived powders. GA contains a trace amount of protein (2%) which can be covalently bound to the carbohydrate group in MD, forming a sound barrier for protecting core materials. In addition, both protein and GA also have film-forming properties. These factors may explain the good bioactive preserving properties of GA when used in combination with MD [20,25]. Furthermore, it is also possible for the hydroxyl moieties in the phenolic compounds to form hydrogen bonds with the trace protein in GA, which would further reinforce the protection of phenolic in the microcapsules, thereby enhancing the TPC retention [43]. Maltodextrin also provided promising results, as observed in the aqueous and ethanol extract powders (84.69% and 75.38%, respectively). These values were reasonable and comparable to the retention of anthocyanins (85%) in blackberry pulp powder [44] and *p*-hydroxybenzoic acid in the majority of mandarin powder samples (81.5–105.8%) [21], though lower than that of TPC retention in cranberry powder (138–216%) [20].

Comparing the extracts, TPC retention in the ethanol extract powders was significantly lower (*p* < 0.05) than the aqueous extract powders (Figure 2B). This could be ascribed to the different chemical compositions of the two extracts. The phenolic compounds in ethanol extract could be more sensitive to heat during spray drying [4], while the aqueous extract also contains polysaccharides [6,12] that might potentially protect phenolics to a certain extent. The lowest TPC retention value (56.23%) was observed for the powder of ethanol extract microencapsulated by MD and GA in 1:1 ratio (EE-MD/GA-2). The more wrinkled particle surface of the EE-MD/GA-2 sample (Figure 1, row B and Column b) also pointed to a relatively higher total surface area than the aqueous extract powders, which could be linked to the lower encapsulation efficiency of phenolic compounds. In addition, the loss of phenolic compounds could also be attributed to their thermal degradation during spray-drying treatment [45].

Figure 2C–F show the antioxidant activities of spray-dried powders and their retentions after spray drying. Comparing the *AOC* of original extracts without encapsulation, the FRAP assay showed significantly higher values (*p* < 0.05) than those from the DPPH assay (i.e., 567.61 vs. 447.54 µM TE/g dw for aqueous extracts and 688.23 vs. 593.87 µM TE/g dw for ethanol extracts). As two assays have different reaction mechanisms, it is necessary to consider both when evaluating the antioxidant capacities, and this has been a practice in many previous studies [11,29,37]. Both DPPH and FRAP assays have similar *AOC* ranges for powder products (Figure 2C,E), i.e., from 331.69 to 486.72 and from 354.43 to 513.49 µM TE/g dw, respectively. These results were positively correlated with the TPC of most powders, indicating a significant contribution of phenolic compounds to the antioxidant properties. An exception was observed for the EE-MD/GA-2 and WE-MD/GA-3 samples; the EE-MD/GA-2 sample has significantly (*p* < 0.05) higher DPPH and FRAP antioxidant activities (444.63 and 480.85 µM TE/g dw, respectively) than the WE-MD/GA-3 sample (331.69 and 354.43 µM TE/g dw), but their TPC values had no statistical significance (*p* > 0.05). The increased *AOC* as displayed by the EE-MD/GA-2 sample might be attributed to its Maillard reaction products that showed antioxidant potential, such as 5-hydroxymethylfurfural [46]. After spray drying, the retention of *AOC* as analyzed by DPPH and FRAP assays varied from 75.27–94.42% and 71.18–92.52% for both extracts, respectively (Figure 2D,F). The aqueous extract encapsulated by the GA-MD mixture of 1:1 showed the highest *AOC* retention of 94.42%. The possible structural changes, degradation, re-synthesis and polymerization of phenolic compounds induced by the heat treatment may be responsible for the reduced antioxidant activities after spray drying [35].

### 3.4. Individual Phenolic Compounds in C. paliurus Powders 

An HPLC-DAD system was used to analyze and quantitate individual phenolic compounds in the resulting powders to investigate the effect of wall materials on phenolic retention and to clarify the suitable sample type (aqueous or ethanol extract) for preparing the *C. paliurus* microcapsules. Table 4 showed the concentrations of 14 major phenolic compounds (seven each for phenolic acids and flavonols) in *C. paliurus* extracts before and after spray drying.

Chlorogenic acid remained predominant in both original extracts and spray-dried powders, accounting for 51–54% of the total quantified phenolic components (33.06–36.32 mg/g dw for powder samples; 40.8 and 45.5 mg/g dw for aqueous and ethanol extracts, respectively). The ethanol extract contained 23.52 mg/g dw chlorogenic acid, significantly higher than the aqueous one (21.96 mg/g dw). The *C. paliurus* powders contained 16.94–17.38 mg/g dw of chlorogenic acid, with the highest value found in the WE-MD/GA2 sample (18.55 mg/g dw). Quercetin-3-*O*-glucuronide was the second major phenolic component, occupied approximately 17% of the total in powder samples. The concentrations of quercetin-3-*O*-glucuronide in the original aqueous and ethanol *C. paliurus* extracts were 4.13 and 4.88 mg/g dw, respectively (Table 4), which was much higher than the findings of Zhou et al. (0.2–3.7 mg/g) [11]. This might be attributed to the plant diversity of *C. paliurus*, as the phenolic profiles of *C. paliurus* were found to be quantitatively and qualitatively different across geographical location [11]. The other major phenolic components in the powder samples included quercetin-3-*O*-rhamnoside, 5-*O*-caffeoylquinic acid, myricetin-3-*O*-galactoside, and 1,3-dicaffeoylquinic acid, with proportions of approximately 8.4%, 6.6%, 5.1%, and 3.1%, respectively. The total quantitative contribution of the remaining eight phenolic compounds was less than 10%, which included kaempferol-3-*O*-glucoside (0.73–0.95 mg/g), kaempferol-3-*O*-rhamnoside (0.71–1.06 mg/g), quercetin-3-*O*-glucoside (0.53–0.80 mg/g), 4,5-dicaffeoylquinic acid (0.36–0.51 mg/g), quercetin-3-*O*-galactoside (0.21–0.32 mg/g), 1,5-dicaffeoylquinic acid (0.15–0.17 mg/g), 3,4-dicaffeoylquinic acid (0.13–0.16 mg/g), and caffeic acid (0.11–0.16 mg/g).

**Figure 3:**
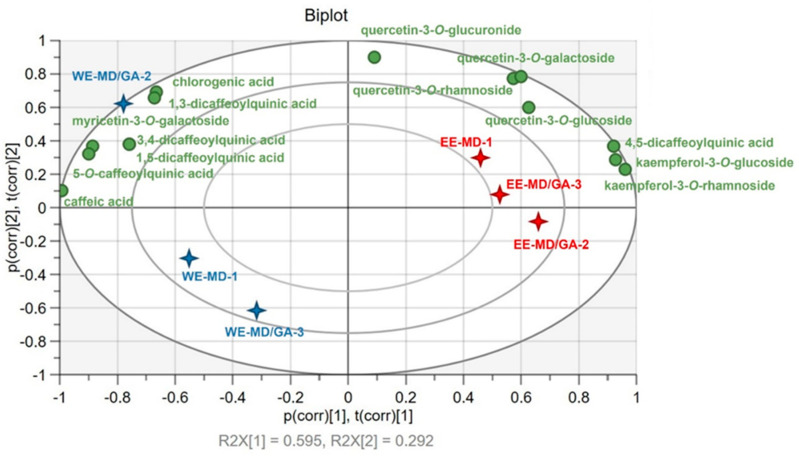
PCA biplot showing the distribution of phenolic compounds in different spray-dried *C. paliurus* powders; WE, aqueous extract; EE, ethanol extract; MD, maltodextrin (10–13 DE); GA, gum acacia. The numbers indicate the wall materials ratio; 1 = only MD, 2 = MD:GA of 1:1 ratio, 3 = MD:GA of 1:3 ratio.

To further visualize the distribution of individual phenolic compounds among powder samples, unsupervised PCA analysis was performed using SIMCA 16 software and the plot is shown as Figure 3. The first two principal components explained a total of 88.7% of variables (59.5% and 29.2%, respectively). The PCA Biplot showed a clear separation between powders derived from different *C. paliurus* extracts. For example, powders of the aqueous extract were located on the left side of the plot, whereas ethanol extract powders were on the right with high loadings of most flavonols such as kaempferol-3-*O*-rhamnoside, kaempferol-3-*O*-glucoside, quercetin-3-*O*-glucoside, quercetin-3-*O*-rhamnoside, etc. Noteworthy, the aqueous extract of *C. paliurus* encapsulated by MD-GA blends of 1:1 (i.e., the WE-MD/GA-2 sample) showed high correlations with one flavonol (myricetin-3-*O*-galactoside) and all phenolic acids except 4,5-dicaffeoylquinic acid.

The distribution of phenolic compounds among powder samples was related to their initial concentrations in the extracts as shown in Table 4. Zhou et al. found that the extraction solvent applied significantly influenced the bioactive profiles of *C. paliurus* extracts [11]. They reported that water was an efficient solvent for extracting phenolic acids, especially 3-*O*-caffeoyluinic acid and 4-*O*-caffeoyluinic acid, while the ethanol yielded high concentrations of triterpenoid and flavonoids [11]. We also observed solvent-specificity towards different phenolic compounds. From Table 4, a significantly higher number of flavonols were extracted by the ethanol than the aqueous solution, with the only exception being that for myricetin-3-*O*-galactoside (aqueous, 1.24 mg/g dw; ethanol, 0.96 mg/g dw). For some phenolic acids, the ethanol solution exhibited higher efficiency than the aqueous solution, such as chlorogenic (35.52 vs. 21.96 mg/g dw), 3,4-dicaffeoylquinic (0.33 vs. 0.24 mg/g dw), and 4,5-dicaffeoylquinic (1.40 vs. 0.96 mg/g) acids. On the other hand, other phenolic acids e.g., 5-*O*-caffeoylquinic, caffeic, 1,3-dicaffeoylquinic, and 1,5-dicaffeoylquinic acids, showed no significant quantitative differences in the aqueous and ethanol extracts. The discrepancy between our results and those reported by Zhou in phenolic acid extraction might be due to the concentration of ethanol used for extraction, i.e., 30% ethanol in the current study vs. 70% in the study of Zhou et al. [11], which affected the polarity of the solvent, and consequently the compound solubility and extraction efficiency.

### 3.5. Retention of Individual Phenolic Compounds after Spray Drying

The hot air applied during spray drying could induce changes to the phenolic compounds. Individual phenolics showed different rates of compound retention, as shown in Table 4. Two flavanols (i.e., quercetin-3-*O*-glucuronide and myricetin-3-*O*-galactoside) were retained at above 100% (115.63–152.6%) in all samples after drying. Similarly, in a previous study, we have found that the contents of myricetin-3-O-galactoside and quercetin-3-galactoside in the spray-dried powers were significantly higher than that of freshly prepared powers (302.0 vs. 74.3 µg/g and 479.5 vs. 205.5 µg/g, respectively) after 8 weeks’ storage at 45 °C [20]. This indicated that some flavonols in the core material might change under high temperatures even after encapsulation. As for the spray-drying process, a shell was gradually formed at the droplet surface, during which the core material suffers thermal treatment up to 180 °C. This offers the possibility of increased contents of quercetin-3-*O*-glucuronide and myricetin-3-*O*-galactoside in the resulting powders. Zhang et al. [20] thought that the above compounds might be released from the phenolic polymers or transformed from other unstable glycosides. Quercetin arabino-furanoside and myricetin arabinoside are less stable than other glycosides and, thus, could be more susceptible to thermal degradation during processing. For example, only 24% and 22% of quercetin arabino-furanoside and myricetin arabinoside were retained in cranberry juices pasteurized at 90 °C for 10 min [47]. Similarly, an accelerated storage of apple juices at 70–100 °C has also confirmed the stability of different glycosides of quercetin. The order of quercetin glycosides stability was found as quercetin galactoside > quercetin glucoside > quercetin arabinoside [48]. Therefore, during the spray-drying process, flavonol arabinoside and arabino-furanoside could be degraded at high temperatures and transformed into more stable chemical forms, i.e., galactoside and glucuronide. These arguments offer explanations for the increased contents of myricetin-3-*O*-galactoside and quercetin-3-*O*-glucuronide after spray drying, as shown in Table 4. However, further research would be necessary to elucidate and confirm this phenomenon, and also investigate the effectiveness of wall materials to protect these unstable flavonol glycosides.

The maximum retention levels obtained for 5-*O*-caffeoylquinic acid, chlorogenic acid, quercetin-3-*O*-glucoside, and 1,3-dicaffeoylquinic acid were 93.77%, 84.56%, 82.06%, and 78.36%, respectively. Chlorogenic acid could be transformed into 5-*O*-caffeoylquinic acid when heated between 125 to 225 °C [49]. Since the *C. paliurus* powders were prepared at an inlet temperature of 180 °C, this conversion might be active in all the spray-dried powder samples, explaining the lower recovery value of chlorogenic acid (72.80–84.56%) than that of the 5-*O*-caffeoylquinic acid (83.86–93.77%). Relatively low retention rates were observed for quercetin-3-*O*-rhamnoside, 3,4-dicaffeoylqunic acid, kaempferol-3-*O*-glucoside, and 1,5-dicaffeoylqunic acid in the powders, with the highest attainment found in the WE-MD/GA-2 sample (*p* < 0.05). Other phenolic compounds have retentions of below 60%, with caffeic acid being the least recovered phenolic (32.80%) in the EE-MD/GA-2 sample.

Despite the fact that the individual phenolic content and recovery varied among samples, significantly higher retention of all the 14 phenolic compounds, especially those widely reported in *C. paliurus* (i.e., chlorogenic acid, quercetin-3-*O*-glucuronide, and 5-*O*-diffeolyquic acid), were significantly higher (*p* < 0.05) in the WE-MD/GA-2 sample. Overall, this sample also exhibited high retention of TPC. The results indicate that microencapsulation of aqueous extract of *C. paliurus* using a combination of MD and GA at 1:1 ratio could be a promising way to protect the phenolic compounds of *C. paliurus*.

## 4. Conclusions

The present study has provided insights into the microencapsulation of *C. paliurus* aqueous- and ethanolic-extracts using a mono-fluidic-jet spray drying technique. The powders produced were uniform in particle sizes with dented and marginally spherical-shaped microstructures. They were bright yellowish to brown colors, resembling the typical color of most tea-derived products. Their solubility in water was excellent, ranging from 88.81 and 99.12%. In addition, the powders have good storage stability, as reflected by their low moisture content (4.09 to 6.64%), water activity (0.11 to 0.19) and hygroscopicity. The phenolic compounds of *C. paliurus* were well-protected in the spray-dried powders, with the highest retention being > 94%. The individual phenolic profile, TPC and *AOC* results have further proven the preservation of phenolic compounds after spray drying. A combination of MD and GA at a ratio of 1:1 gave the best retention of phenolic compounds from the aqueous extract. Overall, the current study demonstrated a promising way to protect the phenolic and antioxidant capacities of *C. paliurus* extracts. The study could serve as a reference for future research and commercial scale processing to produce novel functional food powder with good physicochemical properties and health benefits from *C. paliurus*. Transforming the *C. paliurus* extracts into a versatile powder format could provide a window of opportunity for its application in various functional food products for a wider population.

## Figures and Tables

**Table 1:** Wall material formulations for spray-drying.

No.	Core (Extract)	Sample Abbreviations	Wall Materials (%) ^a^	
			MD	GA
1	WE	WE-MD-1	100	0
2	WE	WE-MD/GA-2	50	50
3	WE	WE-MD/GA-3	75	25
4	EE	EE-MD-1	100	0
5	EE	EE-MD/GA-2	50	50
6	EE	EE-MD/GA-3	75	25

WE, aqueous extract; EE, ethanol extract; MD, maltodextrin (10–13 DE); GA, gum acacia. ^a^ The composition of wall materials; core to wall material used in the current study was 1:3 (w:w).

**Table 2:** Physical properties of spray-dried *C. paliurus* extracts.

Properties	WE-MD-1	WE-MD/GA-2	WE-MD/GA-3	EE-MD-1	EE-MD/GA-2	EE-MD/GA-3
Moisture Content (%)	4.09 ± 0.23 ^a^	4.45 ± 0.21 ^a^	4.73 ± 0.78 ^a^	4.63 ± 0.12 ^a^	5.95 ± 0.77 ^b^	6.64 ± 1.16 ^b^
Water Activity	0.14 ± 0.01 ^bc^	0.18 ± 0.00 ^d^	0.19 ± 0.02 ^d^	0.11 ± 0.00 ^a^	0.13 ± 0.02 ^b^	0.16 ± 0.01 ^c^
Hygroscopicity (g moisture/100 g solids)	3.14 ± 0.40 ^b^	3.00 ± 0.20 ^b^	3.70 ± 0.92 ^b^	2.07 ± 0.34 ^a^	2.07 ± 0.25 ^a^	2.16 ± 0.78 ^a^
Solubility (%)	99.12 ± 0.72 ^d^	94.65 ± 1.40 ^c^	92.04 ± 1.68 ^b^	88.81 ± 1.15 ^a^	89.85 ± 3.11 ^ab^	89.83 ± 1.42 ^ab^
Bulk Density (g/mL)	0.37 ± 0.01 ^c^	0.38 ± 0.01 ^d^	0.38 ± 0.02 ^cd^	0.32 ± 0.01 ^ab^	0.33 ± 0.01 ^b^	0.30 ± 0.00 ^a^

Different letters (^a,b,c,d^) in the same row indicate significant differences at the level of *p* < 0.05. WE, aqueous extract; EE, ethanol extract; MD, malto-dextrin (10–13 DE); GA, gum acacia. The numbers indicate the wall materials ratio; 1 = only MD, 2 = MD:GA of 1:1 ratio, 3 = MD:GA of 1:3 ratio.

**Table 3:** Color attributes and appearance of spray-dried *C. paliurus* extracts.

Samples	Colour Attributes			Powder Appearance
	L*	A*	B*	
WE-MD-1	60.23 ± 0.84 ^a^	11.49 ± 0.15 ^a^	19.07 ± 0.45 ^d^	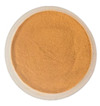
WE-MD/GA-2	59.95 ± 1.53 ^a^	11.56 ± 0.15 ^a^	17.90 ± 0.52 ^c^	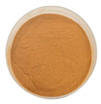
WE-MD/GA-3	59.75 ± 1.14 ^a^	11.65 ± 0.07 ^a^	18.90 ± 0.39 ^d^	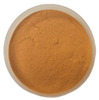
EE-MD-1	59.23 ± 0.63 ^a^	13.25 ± 0.19 ^c^	17.12 ± 0.41 ^b^	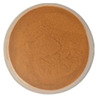
EE-MD/GA-2	59.61 ± 0.94 ^a^	12.94 ± 0.39 ^b^	14.82 ± 0.94 ^a^	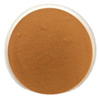
EE-MD/GA-3	60.52 ± 0.62 ^a^	13.68 ± 0.13 ^d^	15.42 ± 0.26 ^a^	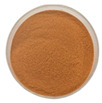

Different letters (^a,b,c,d^) in the same column indicate significant differences at the level of *p* < 0.05. WE, aqueous extract; EE, ethanol extract; MD, maltodextrin (10–13 DE); GA, gum acacia. The numbers indicate the wall materials ratio; 1 = only MD, 2 = MD:GA of 1:1 ratio, 3 = MD:GA of 1:3 ratio.

**Table 4:** The content of major phenolic compounds in powders and their retentions after spray drying.

Phenolic Compounds	Content (mg/g dw)							
	WE	EE	WE-MD-1	WE-MD/GA-2	WE-MD/GA-3	EE-MD-1	EE-MD/GA-2	EE-MD/GA-3
quercetin-3-*O*-glucuronide	4.13 ± 0.01 ^A^	4.88 ± 0.03 ^B^	5.73 ± 0.26 ^a^	6.26 ± 0.04 ^b^	5.75 ± 0.15 ^a^	5.98 ± 0.02 ^ab^	6.02 ± 0.03 ^ab^	6.05 ± 0.06 ^ab^
R (%)			134.06 ± 6.15 ^bc^	152.01 ± 0.84 ^d^	138.68 ± 3.72 ^c^	123.25 ± 0.38 ^a^	122.54 ± 0.56 ^a^	127.55 ± 1.22 ^ab^
myricetin-3-*O*-galactoside	1.24 ± 0.01 ^B^	0.96 ± 0.00 ^A^	1.68 ± 0.06 ^d^	1.84 ± 0.01 ^e^	1.44 ± 0.03 ^c^	1.47 ± 0.07 ^cd^	1.31 ± 0.03 ^ab^	1.24 ± 0.05 ^a^
R (%)			128.80 ± 4.62 ^ab^	149.87 ± 0.70 ^cd^	115.63 ± 2.37 ^a^	152.68 ± 6.82 ^d^	136.65 ± 2.66 ^bc^	136.92 ± 4.92 ^bc^
5-*O*-caffeoylquinic acid	2.45 ± 0.01 ^A^	2.48 ± 0.00 ^A^	2.15 ± 0.02 ^bc^	2.30 ± 0.00 ^d^	2.17 ± 0.00 ^c^	2.09 ± 0.04 ^ab^	2.07 ± 0.01 ^a^	2.06 ± 0.02 ^a^
R (%)			86.10 ± 0.90 ^ab^	93.77 ± 0.09 ^c^	89.04 ± 0.11 ^bc^	83.86 ± 1.63 ^a^	83.44 ± 0.27 ^a^	86.20 ± 0.96 ^ab^
chlorogenic acid	21.96 ± 0.03 ^A^	23.52 ± 0.04 ^B^	17.38 ± 0.02 ^a^	18.55 ± 0.08 ^b^	16.93 ± 0.38 ^a^	17.16 ± 0.23 ^a^	17.14 ± 0.38 ^a^	16.94 ± 0.07 ^a^
R (%)			76.54 ± 0.08 ^ab^	84.56 ± 0.35 ^c^	77.00 ± 1.71 ^b^	72.80 ± 0.97 ^a^	73.01 ± 1.63 ^a^	75.23 ± 0.30 ^ab^
quercetin-3-*O*-glucoside	0.75 ± 0.01 ^A^	0.98 ± 0.01 ^B^	0.62 ± 0.03 ^b^	0.63 ± 0.02 ^bc^	0.53 ± 0.01 ^a^	0.80 ± 0.03 ^d^	0.66 ± 0.01 ^bc^	0.71 ± 0.03 ^c^
R (%)			78.94 ± 3.99 ^bc^	81.90 ± 3.11 ^c^	71.42 ± 1.18 ^ab^	82.06 ± 2.64 ^c^	66.44 ± 1.01 ^a^	73.14 ± 2.85 ^ab^
1,3-dicaffeoylquinic acid	1.41 ± 0.05 ^A^	1.61 ± 0.00 ^A^	1.06 ± 0.01 ^a^	1.14 ± 0.01 ^b^	1.06 ± 0.01 ^a^	1.06 ± 0.02 ^a^	1.03 ± 0.01 ^a^	1.07 ± 0.02 ^a^
R (%)			69.78 ± 0.90 ^c^	78.15 ± 0.64 ^d^	78.36 ± 0.48 ^d^	66.16 ± 1.49 ^ab^	63.58 ± 0.51 ^a^	68.02 ± 1.33 ^bc^
quercetin-3-*O*-rhamnoside	3.91 ± 0.01 ^A^	4.81 ± 0.01 ^B^	2.76 ± 0.05 ^a^	3.00 ± 0.04 ^b^	2.74 ± 0.10 ^a^	2.99 ± 0.01 ^b^	3.03 ± 0.01 ^b^	3.02 ± 0.04 ^b^
R (%)			68.46 ± 1.33 ^c^	76.50 ± 0.94 ^d^	70.23 ± 2.64 ^c^	62.17 ± 0.15 ^a^	62.95 ± 0.26 ^a^	64.94 ± 0.77 ^ab^
kaempferol-3-*O*-glucoside	1.04 ± 0.01 ^A^	1.45 ± 0.02 ^B^	0.74 ± 0.00 ^a^	0.73 ± 0.00 ^a^	0.73 ± 0.02 ^a^	0.95 ± 0.03 ^c^	0.89 ± 0.02 ^b^	0.90 ± 0.01 ^bc^
R (%)			73.11 ± 0.30 ^b^	69.65 ± 0.39 ^b^	70.60 ± 1.43 ^b^	64.59 ± 1.78 ^a^	62.00 ± 1.55 ^a^	63.59 ± 0.97 ^a^
1,5-dicaffeoylquinic acid	0.25 ± 0.01 ^A^	0.27 ± 0.00 ^A^	0.17 ± 0.00 ^b^	0.17 ± 0.00 ^b^	0.15 ± 0.01 ^a^	0.16 ± 0.01 ^ab^	0.14 ± 0.00 ^a^	0.15 ± 0.00 ^ab^
R (%)			59.07 ± 0.05 ^a^	68.18 ± 0.71 ^b^	57.83 ± 2.62 ^a^	60.75 ± 2.63 ^a^	54.18 ± 2.17 ^a^	58.43 ± 1.22 ^a^
3,4-dicaffeoylquinic acid	0.24 ± 0.00 ^A^	0.33 ± 0.00 ^B^	0.16 ± 0.00 ^cd^	0.16 ± 0.00 ^d^	0.14 ± 0.01 ^abc^	0.15 ± 0.01 ^bcd^	0.13 ± 0.00 ^a^	0.14 ± 0.00 ^ab^
R (%)			55.58 ± 0.81 ^c^	67.49 ± 1.49 ^d^	59.33 ± 3.01 ^c^	46.54 ± 2.00 ^b^	39.55 ± 1.12 ^a^	43.32 ± 0.15 ^ab^
kaempferol-3-*O*-rhamnoside	1.31 ± 0.00 ^A^	1.89 ± 0.01 ^B^	0.71 ± 0.01 ^a^	0.71 ± 0.02 ^a^	0.70 ± 0.05 ^a^	1.00 ± 0.03 ^b^	1.06 ± 0.01 ^b^	0.98 ± 0.00 ^b^
R (%)			53.56 ± 0.68 ^a^	54.42 ± 1.29 ^a^	53.52 ± 3.90 ^a^	53.37 ± 1.69 ^a^	55.94 ± 0.33 ^a^	52.29 ± 0.03 ^a^
quercetin-3-*O*-galactoside	0.52 ± 0.02 ^A^	0.69 ± 0.01 ^B^	0.21 ± 0.01 ^a^	0.28 ± 0.01 ^b^	0.21 ± 0.00 ^a^	0.32 ± 0.01 ^c^	0.28 ± 0.01 ^b^	0.29 ± 0.01 ^bc^
R (%)			40.68 ± 1.62 ^a^	52.80 ± 0.90 ^c^	42.55 ± 0.27 ^ab^	46.08 ± 0.96 ^b^	39.87 ± 1.46 ^a^	42.45 ± 0.91 ^ab^
caffeic acid	0.35 ± 0.01 ^A^	0.34 ± 0.00 ^A^	0.15 ± 0.00 ^cd^	0.16 ± 0.00 ^d^	0.14 ± 0.00 ^c^	0.12 ± 0.01 ^ab^	0.11 ± 0.00 ^a^	0.12 ± 0.01 ^b^
R (%)			41.92 ± 0.52 ^cd^	44.54 ± 0.73 ^d^	38.91 ± 0.51 ^bc^	34.79 ± 2.07 ^ab^	32.80 ± 0.34 ^a^	39.74 ± 1.84 ^bc^
4,5-dicaffeoylquinic acid	0.96 ± 0.00 ^A^	1.40 ± 0.02 ^B^	0.38 ± 0.02 ^a^	0.39 ± 0.00 ^a^	0.36 ± 0.01 ^a^	0.51 ± 0.01 ^b^	0.50 ± 0.01 ^b^	0.50 ± 0.01 ^b^
R (%)			36.22 ± 1.46 ^a^	40.60 ± 0.00 ^b^	37.78 ± 0.59 ^a^	36.84 ±0.60 ^a^	35.54 ± 0.48 ^a^	36.03 ± 0.58 ^a^

Different uppercase letters (^A,B^) indicate that the phenolics content of aqueous and ethanol extracts is significantly different (*p* < 0.05). Different lowercase letters (^a,b,c,d^) in the same row indicates significant differences of phenolic compounds in power samples at the level of *p* < 0.05. WE, aqueous extract; EE, ethanol extract; MD, malto-dextrin (10–13 DE); GA, gum acacia. The numbers indicate the wall materials ratio; 1 = only MD, 2 = MD:GA of 1:1 ratio, 3 = MD:GA of 1:3 ratio.

## Supplementary Materials

The following are available online at https://www.mdpi.com/article/10.3390/foods10122910/s1, Table S1: Calibration curves for the quantitation of individual phenolic compounds, Table S2: TPC, DPPH, and FRAP results of the optimized aqueous (WE) and ethanol extracts (EE).
