# Supplementary material for: Microencapsulation of Cyclocarya paliurus (Batal.) Iljinskaja Extracts: A Promising Technique to Protect Phenolic Compounds and Antioxidant Capacities
Source: Foods. 2021 Nov 24;10(12):2910. doi: 10.3390/foods10122910 (PMC8700588; doi:10.3390/foods10122910)
Supplement: Supplementary file 1 [file foods-10-02910-s001.zip › foods-1461950-supplementary.pdf]

Microencapsulation of *Cyclocarya paliurus* (Batal.) Iljinskaja Extracts: A Promising Technique to Protect Phenolic Compounds and Antioxidant Capacities

Xiao Chen[1], Senghak Chhun[1], Jiqian Xiang[2], Pipat Tangjaidee[1], Yaoyao Peng[1], Siew Young Quek[1,3 *]

[1] School of Chemical Sciences, The University of Auckland, Auckland 1010, New Zealand

[2] Enshi Tujia & Miao Autonomous Prefecture Academy of Agricultural Sciences, Hubei Province, 445002, P.R. China

[3] Riddet Institute, Centre of Research Excellence in Food Research, Palmerston North 4474, New Zealand

Author information

* Corresponding author: Siew Young Quek, Ph.D., Professor

Postal address: Food Science Program, School of Chemical Sciences, The University of Auckland, 23 Symonds ST, Auckland Central, Auckland 1010, New Zealand. *E-mail*: sy.quek@auckland.ac.nz (Siew Young Quek); *Tel*: +64 9 373 7599 (85852); OCIRD: http://orcid.org/0000-0001-5244-9895

Xiao Chen, Ph.D.

Postal address: Food Science Program, School of Chemical Sciences, The University of Auckland, 23 Symonds St, Auckland Central, Auckland 1010, New Zealand. *E-mail*: xche622@aucklanduni.ac.nz; OCIRD: http://orcid.org/0000-0003-4231-6372

Senghak Chhun, MSc

Postal address: Food Science Program, School of Chemical Sciences, The University of Auckland, 23 Symonds St, Auckland Central, Auckland 1010, New Zealand. *E-mail*: schh883@aucklanduni.ac.nz

Jiqian Xiang, Professor

Postal address: Enshi Tujia & Miao Autonomous Prefecture Academy of Agricultural Sciences, No. 517 Shizhou Ave, Enshi city, Hubei Province, 445002, P.R. China. *E-mail*: hmxjq@163.com

Pipat Tangjaidee, Ph.D.

Postal address: Food Science Program, School of Chemical Sciences, The University of Auckland, 23 Symonds St, Auckland Central, Auckland 1010, New Zealand. *E-mail*: ptan226@aucklanduni.ac.nz

Yaoyao Peng, Ph.D

Postal address: Food Science Program, School of Chemical Sciences, The University of Auckland, 23 Symonds St, Auckland Central, Auckland 1010, New Zealand. E-mail: yaoyao.peng@auckland.ac.nz

Supplementary Table S1 Calibration curves for the quantitation of individual phenolic compounds

No.	Phenolic compounds	Detection Wavelength (nm)	Calibration curves	R^2
	Phenolic acids			
1	5-*O*-caffeoylquinic acid	320	y = 39.626x + 0.0956	0.9998
2	chlorogenic acid	320	y = 44.433x + 5.737	0.9999
3	caffeic acid	320	y = 103.45x – 9.4807	0.9994
4	1,3-dicaffeoylquinic acid	320	y = 49.561x + 5.1101	0.9999
5	3,4-dicaffeoylquinic acid	320	y = 73.003x –16.836	0.9922
6	1,5-dicaffeoylquinic acid	320	y = 61.919x – 10.611	0.9973
7	4,5-dicaffeoylquinic acid	320	y = 86.455x – 19.005	0.9952
	Flavonols			
8	myricetin-3-*O*-galactoside	320	y = 23.262x – 2.1524	0.9997
9	quercetin-3-*O*-galactoside	254	y = 65.073x – 0.2055	1
10	quercetin-3-*O*-glucoside	254	y = 35.585x + 6.2344	0.9991
11	quercetin-3-*O*-glucuronide	254	y = 25.762x – 4.9546	0.9999
12	kaempferol-3-*O*-glucoside	254	y = 39.812x – 4.1079	0.9994
13	quercetin-3-*O*-rhamnoside	254	y = 50.328x – 4.2946	0.9999
14	kaempferol-3-*O*-rhamnoside	254	y = 45.728x + 9.2381	0.9999

Supplementary Table S2 TPC, DPPH, and FRAP results of the optimized aqueous (WE) and ethanol extracts (EE)

Extract	TPC (mg GA equivalent/g dw)	Antioxidant activities (μM TE/g dw)	
		DPPH	FRAP
**WE**	65.18 ± 2.66^{A}	447.54 ± 5.88^{A}	567.61 ± 10.98^{A}
**EE**	94.76 ± 3.91^{B}	593.87 ± 30.11^{B}	688.23 ± 20.38^{B}

Different letters (A, B) in the same column indicate significant differences ($p < 0.05$).
